# Deletion of Hepatic FoxO1/3/4 Genes in Mice Significantly Impacts on Glucose Metabolism through Downregulation of Gluconeogenesis and Upregulation of Glycolysis

**DOI:** 10.1371/journal.pone.0074340

**Published:** 2013-08-28

**Authors:** Xiwen Xiong, Rongya Tao, Ronald A. DePinho, X. Charlie Dong

**Affiliations:** 1 Department of Biochemistry and Molecular Biology, Indiana University School of Medicine, Indianapolis, Indiana, United States of America; 2 Department of Cancer Biology, The University of Texas MD Anderson Cancer Center, Houston, Texas, United States of America; Boston University School of Medicine, United States of America

## Abstract

Forkhead transcription factors FoxO1/3/4 have pleiotrophic functions including anti-oxidative stress and metabolism. With regard to glucose metabolism, most studies have been focused on FoxO1. To further investigate their hepatic functions, we generated liver-specific *FoxO1/3/4* knockout mice (LTKO) and examined their collective impacts on glucose homeostasis under physiological and pathological conditions. As compared to wild-type mice, LTKO mice had lower blood glucose levels under both fasting and non-fasting conditions and they manifested better glucose and pyruvate tolerance on regular chow diet. After challenged by a high-fat diet, wild-type mice developed type 2 diabetes, but LTKO mice remained euglycemic and insulin-sensitive. To understand the underlying mechanisms, we examined the roles of SIRT6 (Sirtuin 6) and Gck (glucokinase) in the FoxO-mediated glucose metabolism. Interestingly, ectopic expression of SIRT6 in the liver only reduced gluconeogenesis in wild-type but not LTKO mice whereas knockdown of *Gck* caused glucose intolerance in both wild-type and LTKO mice. The data suggest that both decreased gluconeogenesis and increased glycolysis may contribute to the overall glucose phenotype in the LTKO mice. Collectively, FoxO1/3/4 transcription factors play important roles in hepatic glucose homeostasis.

## Introduction

Mammals have four genes encoding the O subfamily of the Forkhead transcription factors: *FoxO1/3/4/6* [1,2]. Among them, *FoxO1* has been extensively studied. It has been shown that FoxO1 regulates hepatic gluconeogenesis through upregulation of several key genes including phosphoenoylpyruvate carboxykinase (*Pck1*) and glucose 6-phosphatase (catalytic subunit, *G6pc*) [3–12]. Under insulin resistance conditions, FoxO1 becomes less phosphorylated at the inhibitory serine/threonine residues and therefore more active to promote expression of these gluconeogenic genes, which may contribute to hyperglycemia in diabetes [13,14]. This notion is generally supported by the data from overexpression and knockout/knockdown of *FoxO1*. Overexpression of the constitutively active *FoxO1* mutant increases blood glucose levels and leads to impaired glucose and insulin tolerance [11,12]. In contrast, knockout or knockdown of hepatic *FoxO1* lowers blood glucose levels and improves systemic insulin sensitivity in genetic or diet-induced diabetic mouse models [3,4,6,15]. Recently, two mouse genetic studies have reported inconsistent data on the roles of FoxO1 and FoxO3 in glucose metabolism [16,17]. Haeusler and colleagues have shown that a double deletion of hepatic *FoxO1* and *FoxO3* genes in mice has similar effects on blood glucose and glucose tolerance as compared to knockout of the *FoxO1* gene alone [17]. However, Zhang and coworkers have found that FoxO1 and FoxO3 have significant additive effects on glucose homeostasis [16]. Moreover, liver-specific *FoxO1/3/4* knockout mice also manifest lower serum insulin levels and better glucose tolerance as compared to control mice although animal ages are not specified in the report [17]. Additionally, FoxO6 is predominantly expressed in the brain and also has a significant role in hepatic gluconeogenesis [18,19]. However, molecular mechanisms with regard to the collective roles of FoxOs in hepatic glucose metabolism are still elusive. In this work, we attempted to examine the pathophysiological functions of FoxO1/3/4 in glucose metabolism and the underlying mechanisms.

## Materials and Methods

### Animals, blood chemistry, and metabolic analysis

*FoxO1/3/4* floxed mice were generated and genotyped as previously described [20]. To generate liver-specific *FoxO1/3/4* triple knockout mice, the floxed mice were crossed with a line of Albumin-Cre mice (Jackson Lab). Animals were maintained on a mixed genetic background (C57/BL6/129/FVB). Mice were fed either regular chow diet or a high-fat diet (HFD, 60% calories from fat, Harlan Teklad). Adenovirus injections were performed via tail vein as previously described [21]. Blood glucose levels were measured using a glucose meter (Contour from Bayer) under *ad libitum* (non-fasted) or overnight 16-hour fasting conditions. Plasma insulin was measured using a commercial assay kit (ALPCO). Glucose, pyruvate and insulin tolerance tests were performed as previously described [4], with 2 g glucose or pyruvate per kg body weight and 0.75-1 U insulin (humulin R, Lilly) per kg body weight, respectively. Body composition was analyzed by dual-energy X-ray absorptiometry (DEXA). As males and females had similar phenotype, only male data were presented here.

### Ethics statement

All procedures were performed in accordance with the Guide for Care and Use of Laboratory Animals of the National Institutes of Health and were approved by the Institutional Animal Use and Care Committee of Indiana University School of Medicine (study 10322).

### Adenovirus preparation

SIRT6 and GFP overexpression adenoviruses were prepared in an AdEasy system (Agilent) following the manufacturer’s manual. The cloning PCR primers for the human *SIRT6* coding sequence are: SIRT6-forward, 5'-ACTTCCGATATCGCCACCATGTCGGTGAATTACGCGGC-3', and SIRT6-reverse, 5'-AAGGAACTCGAGGCTGGGGACCGCCTTG-3'. *Gck* and *GFP* shRNA adenoviruses were made in a BLOCK-iT system (Invitrogen). The target mRNA sequences are described in the following: *mGck*, 5'-GCTGGTAGAGGAGAATCTTCT-3', and *GFP*, 5'-GCATCAAGGTGAACTTCAAGA-3'.

### Protein analysis

Liver tissue was homogenized in the lysis buffer (50 mM Hepes, pH 7.5, 150 mM NaCl, 10% Glycerol, 1% Triton X-100, 1.5 mM MgCl_2_, 1 mM EGTA, 10 mM Sodium Pyrophosphate, 100 mM Sodium Fluoride, and freshly added 100 µM Sodium Vanadate, 1 mM PMSF, 10 µg/ml Aprotinin, and 10 µg/ml Leupeptin). Proteins were resolved on an SDS-PAGE gel and were transferred to nitrocellulose membrane. The membrane was incubated with the following specific antibodies: SIRT6 (Sigma), Gck and Actinin (Santa Cruz Biotechnology). Protein signals were detected by incubating with HRP-conjugated secondary antibodies and subsequent ECL detection reagents (Thermo, Fisher Scientific).

### RNA analysis

RNA isolation was performed using TRI reagent (Sigma) as described previously [4]. Then cDNA was synthesized using a kit (Applied Biosystems Inc.) and real-time PCR was performed using GoTaq qPCR Mix (Promega). Primer sequences of the mouse genes used in this work are described as follows: *Pck1* forward 5’- AGAAGGAGTACCCATTGAG-3’, *Pck1* reverse 5’- CTGAGGGCTTCATAGACA-3’; *G6pc* forward 5’-ATGGTCACTTCTACTCTTGC-3’, *G6pc* reverse 5’- CAAGATGACGTTCAAACAC-3’; *Gck* forward 5’- AAGGACAGGGACCTGGGTTCCA-3’, *Gck* reverse 5’-TCACTGGCTGACTTGGCTTGCA-3’; *Pklr* forward 5’-TAGGAGCACCAGCATCATTG-3’, *Pklr* reverse 5’- CATCCCTGCCTTGATCATCT-3’; *Pdk2* forward 5’-TGTGGTGAAAGACGCCTATG-3’, *Pdk2* reverse 5’-GTGGCATTGACTTCCTGGAT-3’; *Ppia* forward 5’-CACCGTGTTCTTCGACATCA, *Ppia* reverse 5’- CAGTGCTCAGAGCTCGAAAGT-3’. Real-time PCR data were presented as relative values over an internal control—Ppia.

### Statistical analysis

Data were presented as means ± SEM. Two-tailed unpaired Student’s *t*-test was used to assess the difference between two groups, and *P* < 0.05 was considered as significant.

## Results

### Deletion of FoxO1/3/4 genes in mouse liver significantly alters glucose metabolism

**Figure 1:**
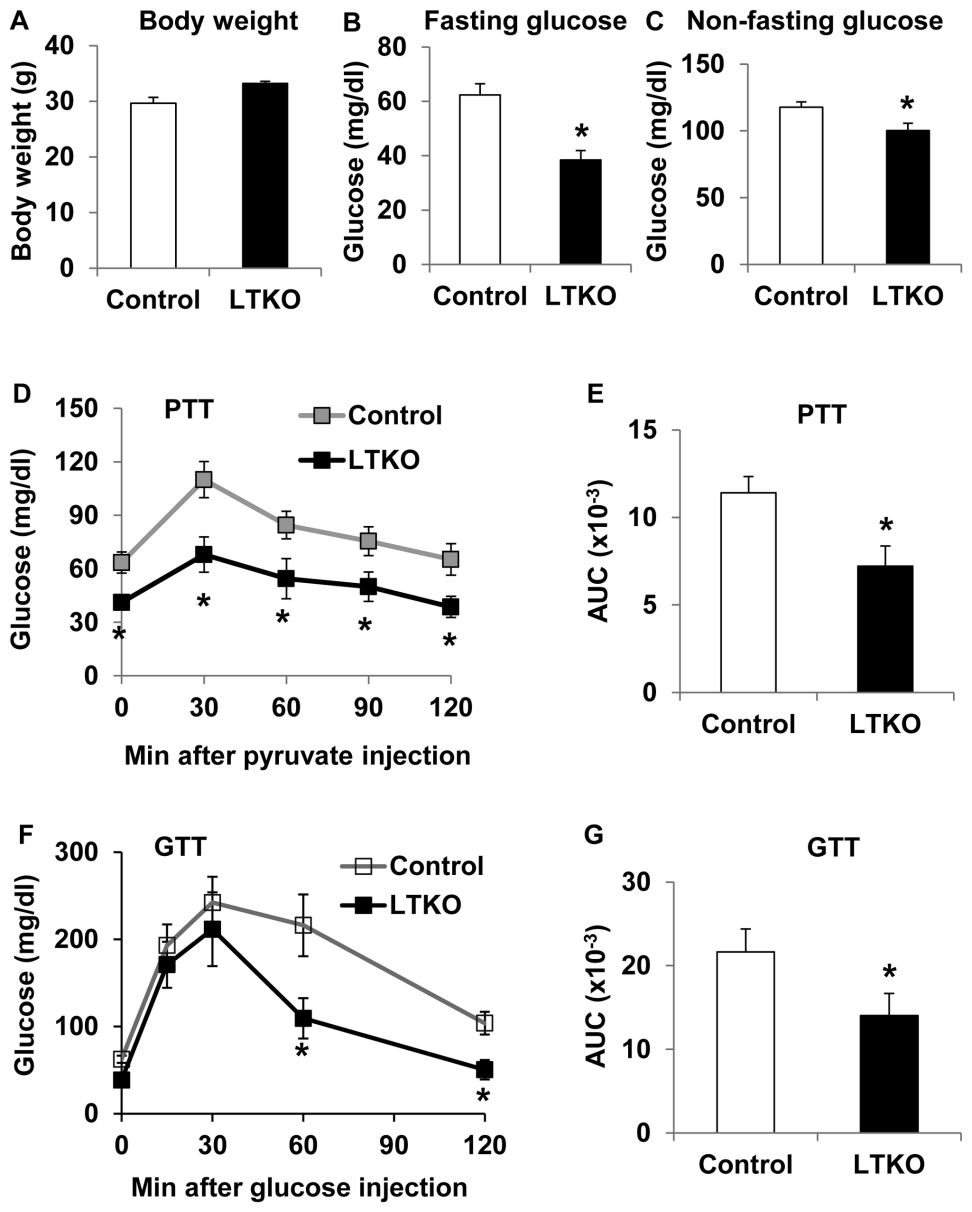
Glucose metabolism in the liver-specific *FoxO1/3/4* knockout mice (LTKO) fed chow diet. (A) Body weight of control and LTKO male mice (n=6) at age of 4 months. (B) Blood glucose levels in 2-month male control and LTKO mice (n=6) after an overnight 16-hour fasting. (C) Non-fasting blood glucose levels in 4-month control and LTKO male mice (n=6). (D, E) Pyruvate tolerance tests (PTT) in 4-month male control and LTKO mice (n=6-7) after an intraperitoneal injection of 2 g pyruvate solution per kg body weight. The areas under the curve (AUC) in the PTT graph were also presented. (F, G) Glucose tolerance tests (GTT) in 3-month male control and LTKO mice (n=6) after an intraperitoneal injection of 2 g glucose solution per kg body weight. The areas under the curve in the GTT graph were also presented. Data represent mean ± SEM. * indicates a significance with *P*<0.05 in control vs. LTKO mice.

Since some previous reports have shown that FoxO1/3/4 have a significant extent of functional redundancy *in vivo* [16,17,20,22], here we investigated their collective roles in glucose homeostasis by deletion of all 3 genes in mouse liver (LTKO) using floxed alleleles and an Albumin-Cre transgene. Although there was no significant difference in body weight between wild-type and LTKO mice (Figure 1A), deletion of *FoxO1/3/4* in the liver resulted in a decrease in blood glucose levels by 38% and 15% in male adult mice under overnight fasted and non-fasted conditions, respectively (Figure 1, B and C). Since FoxO1 has been shown to regulate hepatic gluconeogenesis [3–12,16], we examined this process in control wild-type and LTKO mice using pyruvate tolerance tests, which measure the rate of *de novo* glucose synthesis using pyruvate as a substrate. As expected, after the pyruvate injection, blood glucose rose to a much lower level in the LTKO mice compared to the control mice, and the area under the curve (AUC) was 37% less than that in the control mice (Figure 1, D and E). Glucose tolerance tests were also performed to assess changes in glucose disposal, and the results showed that exogenous glucose was cleared much faster in LTKO mice than that in control mice (Figure 1F). The AUC of the overall glucose tolerance was decreased by 35% in the LTKO mice (Figure 1G).

### Insulin levels are decreased in LTKO mice

**Figure 2:**
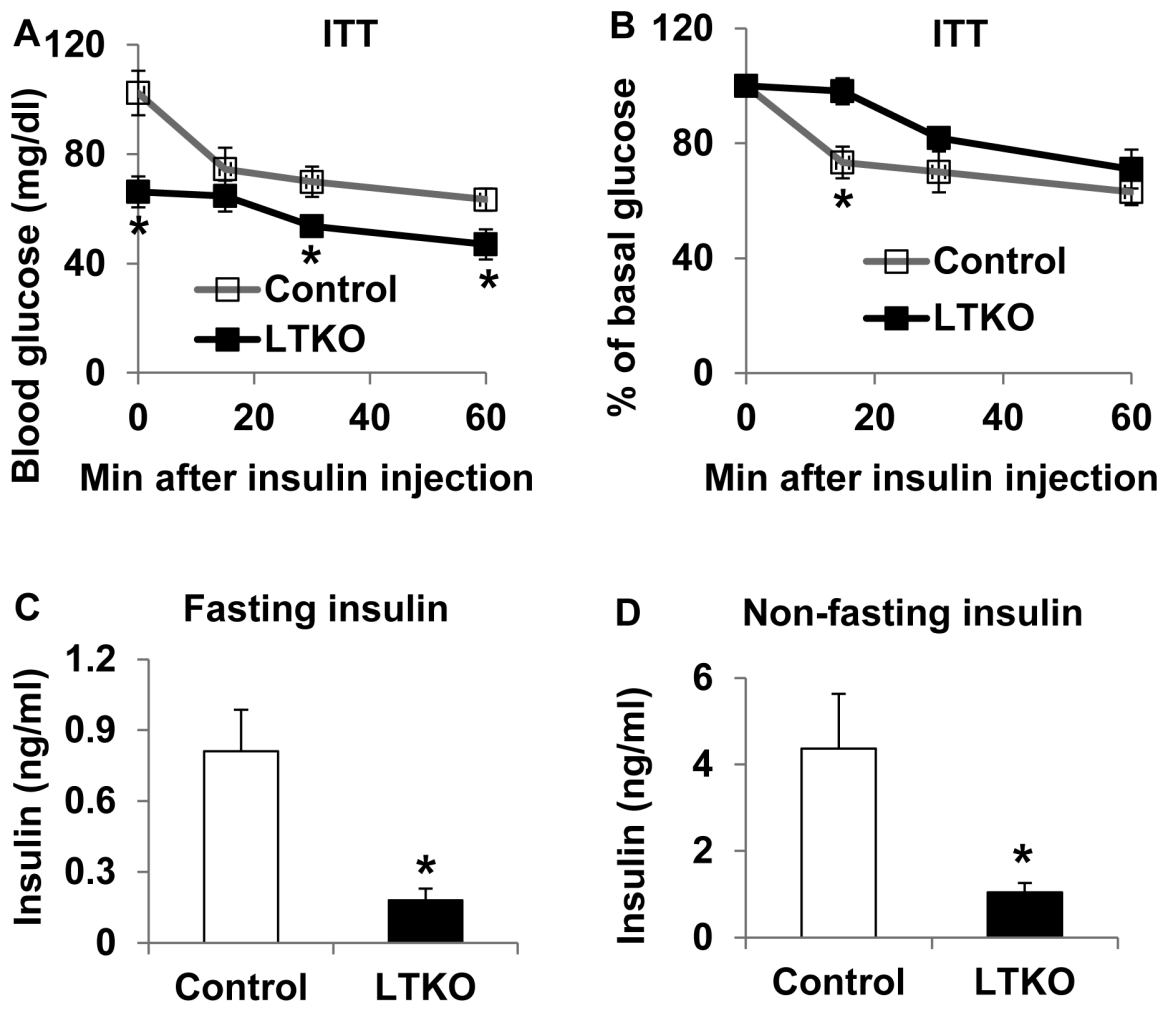
Insulin sensitivity in LTKO mice fed chow diet. (A) Insulin tolerance tests (ITT) in 3-month male control and LTKO mice (n=6) after 3-hour fasting and an intraperitoneal injection of 0.75 U human regular insulin (humulin R, Lilly) per kg body weight. (B) The data in Panel A were replotted as percentage of basal blood glucose as a function of injection time. (C) Plasma insulin levels in 4-month male control and LTKO mice (n=12) after an overnight 16-hour fasting. (D) Plasma insulin levels in 4-month male control and LTKO mice (n=6) under *ad libitum* conditions. Data represent mean ± SEM. * indicates a significance with *P*<0.05 in control vs. LTKO mice.

To assess insulin sensitivity, we first performed insulin tolerance tests in 3-month old mice. Since the basal blood glucose levels were already low in the LTKO mice, an exogenous insulin bolus did not reduce glucose as much as in the control wild-type mice (Figure 2A). This phenomenon could also be seen after glucose levels were normalized to the basal for the ITT data (Figure 2B). In addition, plasma insulin levels were 4 fold lower in the LTKO mice as compared to control wild-type mice under both fasting and non-fasting conditions (Figure 2, C and D).

### Hepatic deficiency of FoxO1/3/4 protects mice from developing high-fat diet-induced diabetes

**Figure 3:**
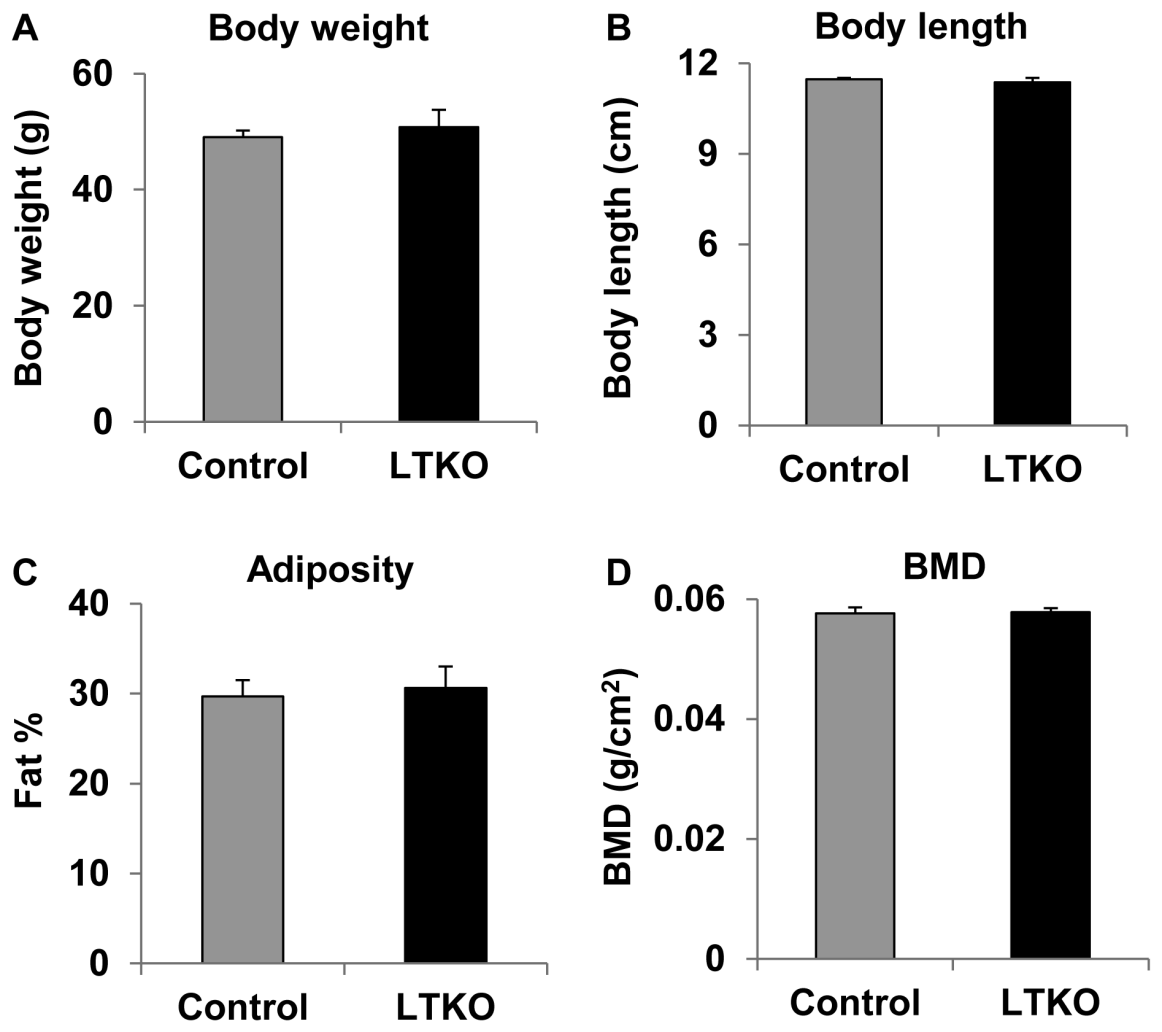
Body composition of LTKO mice fed a high-fat diet. (A, B) Body weight and length measurements of control and LTKO mice (n=6) after a high-fat diet (HFD) treatment for 5 months, respectively. (C, D) Body fat and bone mineral density (BMD) analyses of the above HFD treated mice by DEXA, respectively. Data represent mean ± SEM.

**Figure 4:**
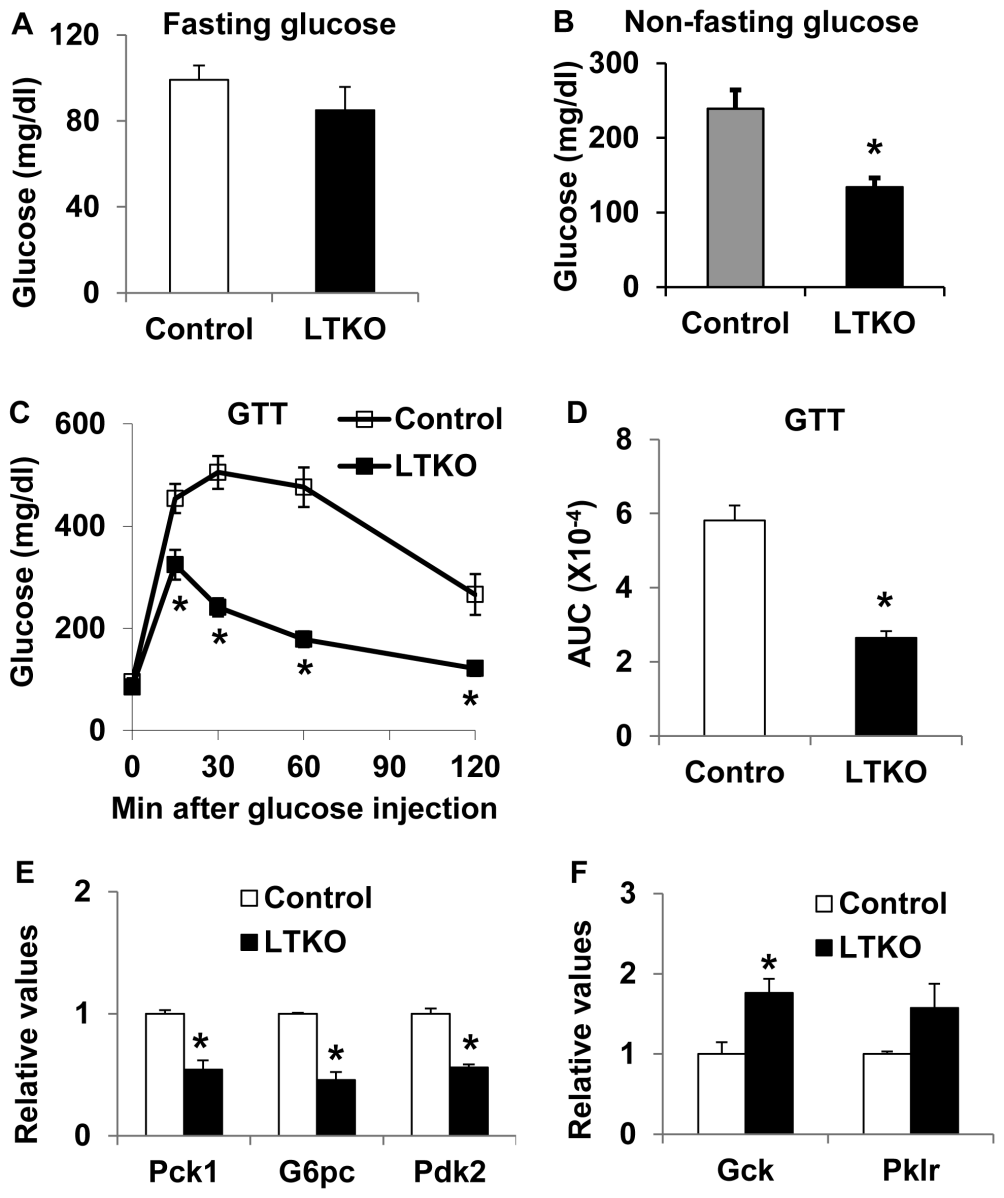
LTKO mice maintain euglycemic and glucose tolerant on a high-fat diet. (A) Fasting glucose levels in 4.5-month male control and LTKO mice (n=8) after the treatment with a high-fat diet for 3.5 months. (B) Non-fasting blood glucose levels in 4-month male control and LTKO mice (n=8) after the treatment with the high-fat diet for 3 months. (C, D) Glucose tolerance tests and the AUC analysis in 4.5-month male control and LTKO mice (n=8) after the treatment with the high-fat diet for 3.5 months, respectively. (E, F) Expression of genes involved in glucose metabolism was analyzed in the liver of control and LTKO mice (n=4) treated with the high-fat diet for 5 months by real-time PCR. *Pck1*, phosphoenoylpyruvate carboxykinase 1; *G6pc*, glucose-6-phosphatase, catalytic; *Pdk2*, pyruvate dehydrogenase kinase 2; *Gck*, glucokinase; *Pklr*, pyruvate kinase, liver and red blood cell type. Data represent mean ± SEM. * indicates a significance with *P*<0.05 in control vs. LTKO mice.

**Figure 5:**
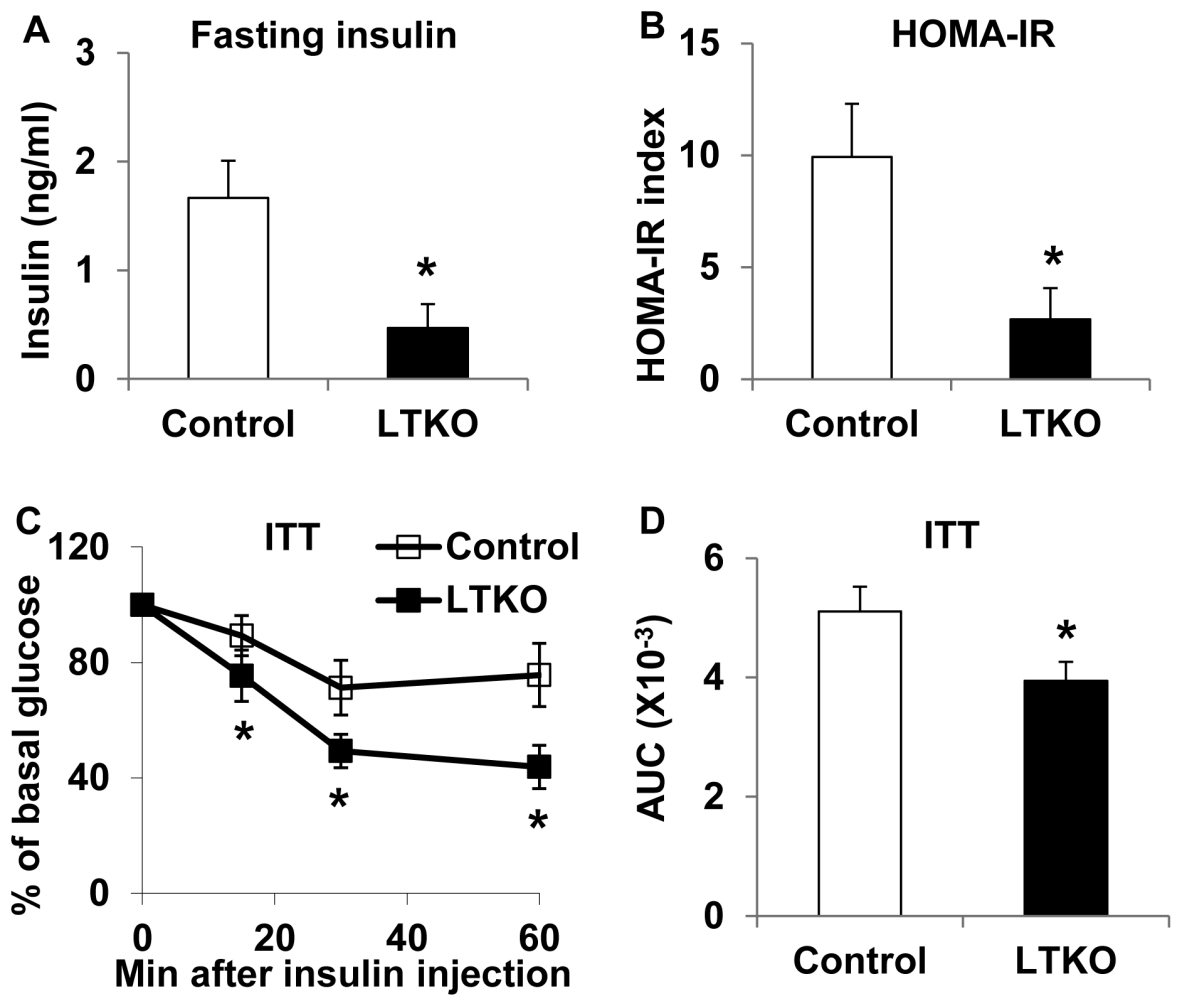
LTKO mice remain insulin-sensitive on a high-fat diet. (A) Fasting plasma insulin levels in 6-month male control and LTKO mice (n=8) after the treatment with a high-fat diet for 5 months. (B) HOMA insulin resistance (IR) index was calculated using the fasting blood glucose and insulin data collected from control and LTKO male mice treated with the high-fat diet for 5 months. (C, D) Insulin tolerance tests and the AUC analysis in 4-month male control and LTKO mice (n=8) using a dose of 1 U insulin per kg body weight after the treatment with the high-fat diet for 3 months, respectively. Data represent mean ± SEM. * indicates a significance with *P*<0.05 in control vs. LTKO mice.

Since LTKO mice had lower glucose levels relative to wild-type mice on regular chow diet, we went on to test whether deletion of hepatic *FoxO1/3/4* might protect mice from developing high-fat diet-induced diabetes. Control wild-type and LTKO mice were fed a high-fat diet (HFD) and they were subsequently monitored for up to 5 months. At the end of the HFD treatment, there was no significant difference in body composition parameters, including body weight, body length, body fat, and bone mineral density between wild-type and LTKO mice (Figure 3, A-D). As early as 3 months after the HFD treatment, the control mice developed hyperglycemia; however, the LTKO mice remained euglycemic under both fasted and non-fasted conditions (Figure 4, A and B). Systemic glucose tolerance in the LTKO mice was much better than that in the control mice during the GTT tests, and the AUC was 55% lower in the LTKO mice (Figure 4, C and D). At molecular levels, expression of gluconeogenic genes including *Pck1*, *G6pc* and *Pdk2* was decreased in the LTKO livers as compared to the controls while expression of the glycolytic gene *Gck* went up (Figure 4, E and F). In addition, fasting insulin levels were 3-fold lower in the LTKO mice as compared to the control mice, and homeostatic model assessment (HOMA) also showed 4-fold decrease in insulin resistance in the LTKO mice (Figure 5, A and B). Moreover, LTKO mice had better insulin tolerance than the control mice and the AUC was decreased by 23% in the LTKO mice (Figure 5, C and D).

### The role of SIRT6 in FoxOs-regulated hepatic gluconeogenesis

**Figure 6:**
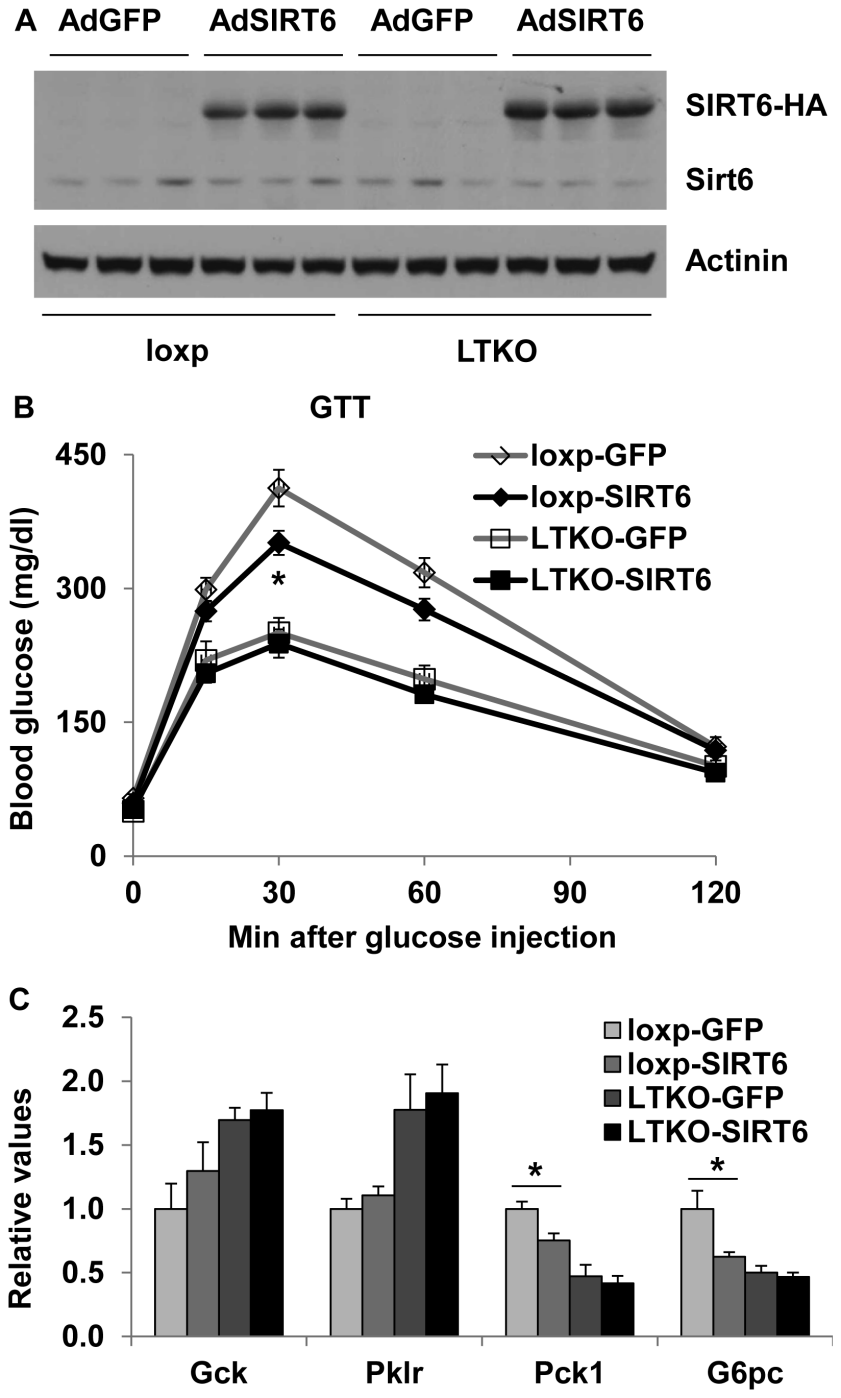
Sirt6 overexpression has no significant effect on glucose toerance in LTKO mice. (A) Sirt6 overexpression was assessed by Western blot analysis in liver lysates from control and LTKO mice injected with SIRT6 or GFP adenoviruses (n=6). (B) Glucose tolerance tests in 4-month-old control and LTKO mice injected with SIRT6 or GFP adenoviruses (n=6). (C) Expression of genes involved in glucose metabolism was analyzed in the livers of SIRT6 or GFP adenovirus infected control and LTKO mice (n=6) by real-time PCR. Data represent mean ± SEM. * indicates a significance with *P*<0.05 between loxp-GFP and loxp-SIRT6 groups.

Previously, SIRT6 has been reported to suppress both hepatic glycolysis and gluconeogenesis through epigenetic regulation of the related genes such as *Gck*, *Pklr*, *Pck1*, and *G6pc* [23,24]. Here we attempted to explore whether SIRT6 might play a role in the FoxO-regulated glucose metabolism. We used adenovirus-mediated gene transfer approaches to specifically overexpress control GFP or human SIRT6 in wild-type or LTKO mouse livers (Figure 6A). Glucose tolerance tests were performed 7 days post-injection. SIRT6 overexpression improved glucose tolerance in the wild-type mice but not LTKO mice (Figure 6B), suggesting that FoxO1/3/4 may be needed for this metabolic regulation by SIRT6. Gene expression analysis revealed that gluconeogenesis (*Pck1* and *G6pc*) but not glycolysis (*Gck* and *Pklr*) genes were suppressed by SIRT6 in the wild-type livers only (Figure 6C).

### The role of hepatic Gck in FoxOs-modulated glucose metabolism

**Figure 7:**
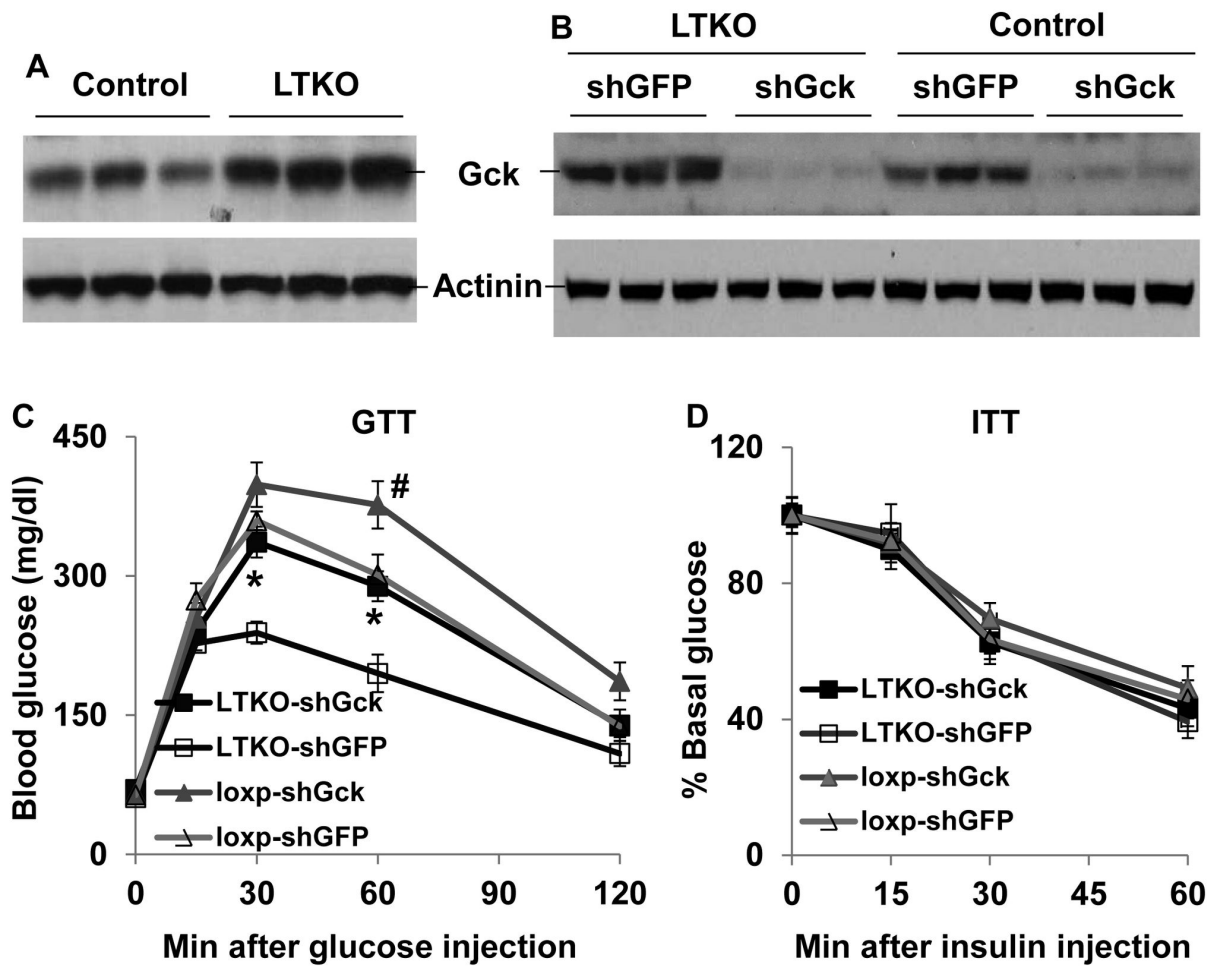
Gck knockdown impairs glucose tolerance in both wild-type and LTKO mice. (A) Gck protein was analyzed in the livers of 3-month-old control and LTKO mice by Western blots. (B) Gck knockdown was assessed by Western blots in liver lysates from control and LTKO mice injected with shGck or shGFP adenoviruses. (C, D) Glucose tolerance tests and insulin tolerance tests in 6-month-old male control and LTKO mice injected with shGck or shGFP adenoviruses (n=5-6), respectively. Data represent mean ± SEM. *, *P*<0.05 between LTKO-shGFP and LTKO-shGck groups; #, *P*<0.05 between loxp-shGFP and loxp-shGck groups.

In addition to gluconeogenesis, FoxOs have been implicated in glycolysis in the liver [12,16,17,25–27]. Indeed, Western blot analysis showed that Gck protein was increased more than 2-fold in the LTKO livers (Figure 7A). To test the extent of the elevated Gck expression to glucose metabolism in LTKO mice, we knocked down hepatic *Gck* gene using adenovirus-mediated shRNAs (Figure 7B). Seven days post-injection, we performed glucose tolerance tests, and the results showed that knockdown of the *Gck* gene led to glucose intolerance in both wild-type and LTKO mice (Figure 7C). Two days later, we also performed insulin tolerance tests. No difference was observed regardless of genotypes or gene knockdown (Figure 7D). These data suggest that Gck mediated hepatic glycolysis also plays a significant role in FoxOs-regulated glucose metabolism.

## Discussion

FoxO family members have been shown to regulate a number of common target genes including those involved in metabolism [3,9,13,17,20–22,28–36]. In this study, we demonstrate that combined deletion of *FoxO1/3/4* exerts a strong impact on hepatic glucose metabolism. LTKO mice manifest lower blood glucose levels under both fasting and non-fasting conditions as compared to control mice. One of the major contributing factors may be the attenuated hepatic gluconeogenesis since pyruvate tolerance is much better in the LTKO mice and expression of gluconeogenic genes including *Pck1*, *G6pc*, and *Pdk2* is lower in the LTKO livers as compared to controls. Additionally, while there is no significant alteration in *Pklr* gene expression, *Gck* gene expression is significantly increased in the LTKO mice. Remarkably, knockdown of hepatic *Gck* gene reduces glucose tolerance in the LTKO mice. These data suggest that increased glycolysis also significantly contributes to the rapid glucose clearance in hepatic FoxO1/3/4 deficient mice. This conclusion is consistent with previous reports using liver-specific *Gck* transgenic and knockout mice [37–42]. Although an increase of the *Gck* gene copy may protect mice from developing severe diabetes [43], long-term overexpression of *Gck* in the liver also causes fatty liver and insulin resistance [44]. With regard to LTKO mice, although they have better insulin sensitivity after 5 months of high-fat treatment, those mice also developed hepatic steatosis as we previously reported [22]. Thus, it is likely that LTKO mice may eventually develop insulin resistance under obesity-prone conditions.

It is also possible that FoxOs may regulate glucose metabolism through their impact on insulin signaling. Previously, it has been shown that constitutively nuclear FoxO1 mutant can enhance Akt (S473) phosphorylation through suppression of the inhibitory pseudokinase *Trib3* gene expression [45]. However, no differences in *Trib3* gene expression and insulin-stimulated Akt (S473) phosphorylation are observed between control and LTKO livers (data not shown), raising a question as to whether Trib3 is involved in the FoxO-regulated glucose metabolism.

Recently, Sirt6 has been implicated in the regulation of hepatic glycolysis and gluconeogenesis [24]. Although expression of *Gck* and *Pklr* genes has been shown to be upregulated in the liver of hepatic Sirt6 knockout mice [24], overexpression of Sirt6 does not suppress *Gck* and *Pklr* gene expression in either wild-type or LTKO livers. This suggests that additional factors may be needed to achieve the suppression of glycolytic genes. Nevertheless, Sirt6 overexpression reduces gluconeogenic gene expression in the liver of wild-type but not LTKO mice, implying that Sirt6 might coordinate with FoxOs in the regulation of gluconeogenesis. PGC-1α, a target gene of FoxO1 and also a coactivator of FoxO1, has been shown to be regulated by SIRT6 through control of the GCN5 acetyltransferase activity [23]. Thus, it is possible that SIRT6 modulates hepatic gluconeogenesis through both PGC-1α and FoxO1.

Significantly, LTKO mice remain euglycemic and insulin-sensitive on high-fat diet for at least 5 months in this study. This phenotype is consistent with a previous report that hepatic deletion of FoxO1 and FoxO3 also improves glucose and insulin tolerance in diabetic db/db mice [16]. Also individually, FoxO1 and FoxO6 have been shown to exert significant impact on glucose metabolism, particularly on hepatic gluconeogenesis. Overexpression of *FoxO1* or *FoxO6* in mouse liver causes elevated fasting blood glucose levels and impaired glucose tolerance [11,12,19]. Conversely, knockdown or knockout of hepatic *FoxO1* or FoxO6 improves glucose homeostasis in some diabetic mouse models [3,4,6,15,19]. Hepatic FoxO1/3/4 knockout mice have been previously shown to develop hypoglycemia at postnatal and adult ages [17]. In that report, the authors have also found that expression of *Pck1* is significantly decreased and *Gck* is increased [17]. In our study, we have investigated the potential mechanisms that lead to altered glucose metabolism upon hepatic deletion of *FoxO1/3/4* genes. We have observed that in addition to *Pck1*, other genes such as *G6pc* and *Pdk2* are also significantly downregulated in the LTKO liver. Our high-fat diet study further confirms that ablation of hepatic *FoxOs* can protect animals from developing hyperglycemia. More importantly, our mechanistic studies using liver-specific *Gck* gene knockdown and *Sirt6* overexpression reveal that both are involved in FoxOs-regulated glucose metabolism. Taken together, our findings suggest an important role of FoxO1/3/4 in the regulation of glucose metabolism under physiological conditions and a potential implication in the pathogenesis of diabetes.
